# Supplementary material for: Synchronization Through Uncorrelated Noise in Excitatory-Inhibitory Networks
Source: Front Comput Neurosci. 2022 Feb 4;16:825865. doi: 10.3389/fncom.2022.825865 (PMC8855529; doi:10.3389/fncom.2022.825865)
Supplement: Supplementary file 1 [file Data_Sheet_1.pdf]

# Supplementary Material

## 1 SUPPLEMENTARY DATA

### 1.1 Figures

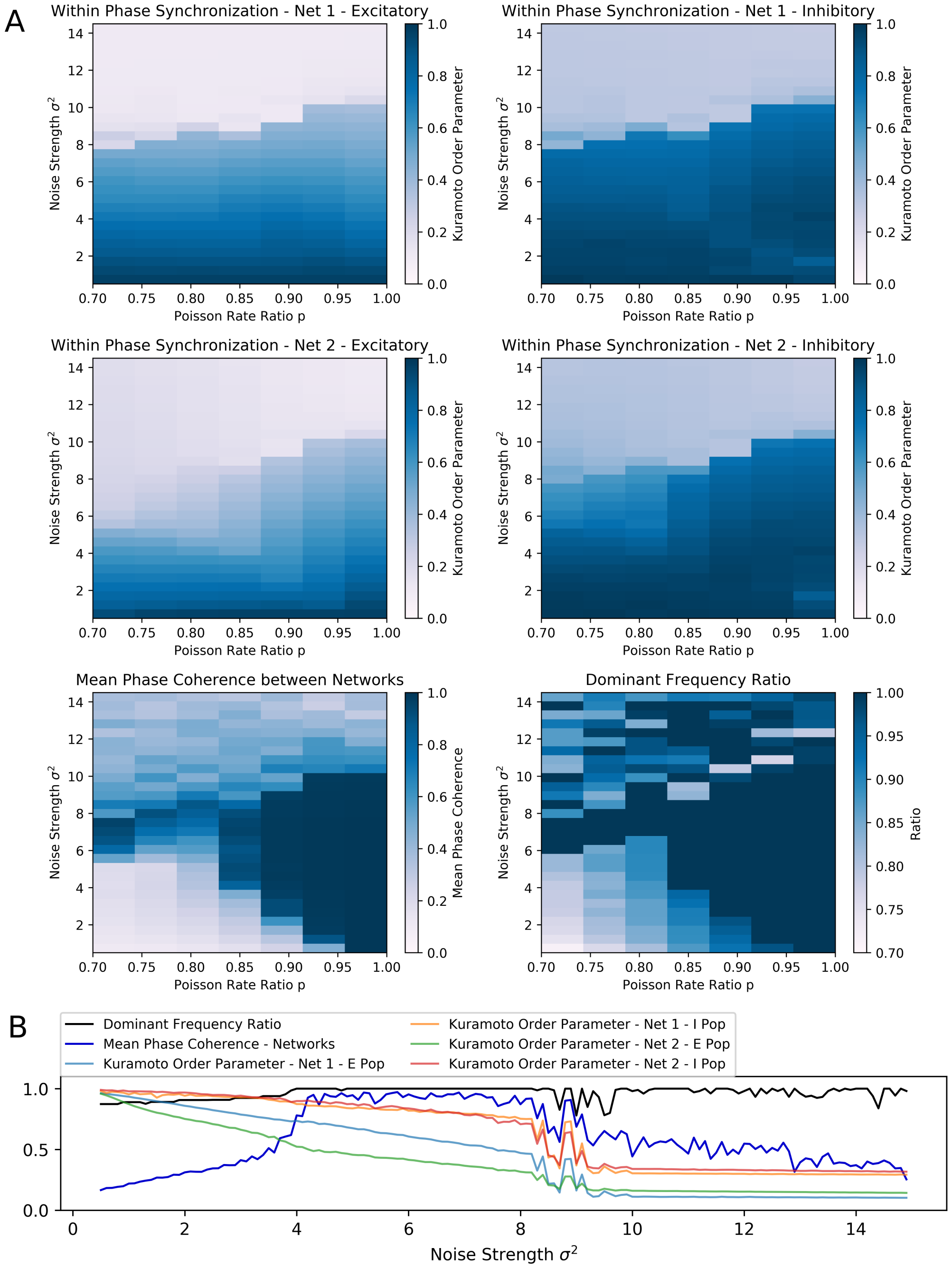


**Figure S1. Scenario 2: Exploration of two interacting all-to-all connected excitatory-inhibitory networks driven by the PING mechanism**
(A) Exploring the within and across network synchronization behavior over different noise strengths $\sigma^2$ and noise frequency ratio $p$ values. (B) One-dimensional explorations over noise strength $\sigma^2$. Noise frequency ratio stayed constant with $p = 0.85$. Range of 0.5 to 15.0 in 0.1 steps with runtime of 3s for each trial.

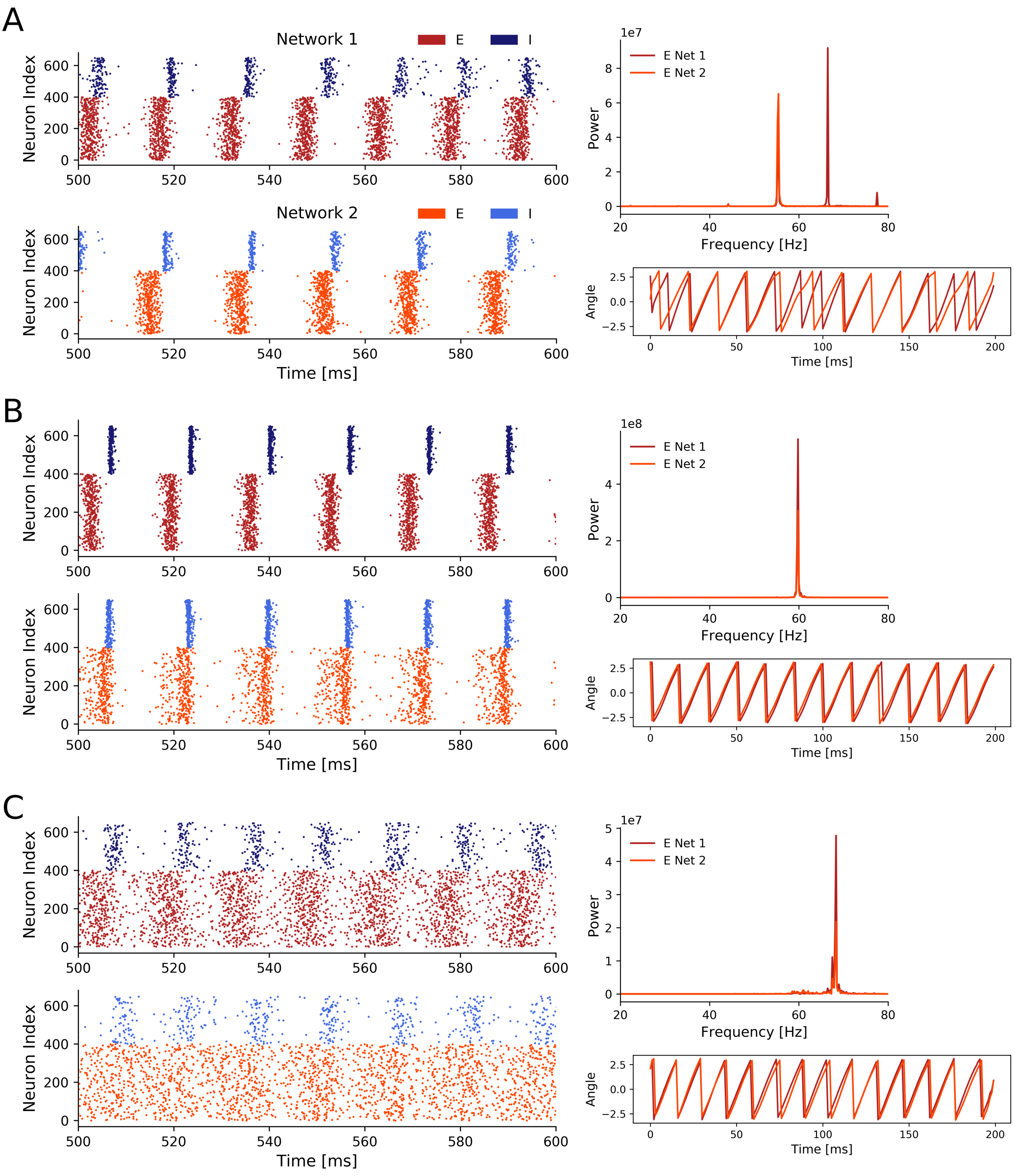


**Figure S2. Three representative states of scenario 3 for a current-based LFP proxy.** Identical analysis to Figure 5 of the main manuscript, however, the LFP signal is calculated as the sum of the absolute value of all excitatory and inhibitory synaptic currents of the excitatory populations. (A) Weak noise and weak inter-network coupling. With $p = 0.85$, $\sigma^2 = 0.7$, $J_{ppei} = 0.03$. (B) Coupling strength was increased until we observed 1:1 frequency entrainment. With $p = 0.85$, $\sigma^2 = 0.7$, $J_{ppei} = 0.07$. (C) Strong noise and weak inter-network coupling. With $p = 0.85$, $\sigma^2 = 4.5$, $J_{ppei} = 0.03$. Only 400 out of 1000 excitatory (red) neurons are displayed in the spike raster plots to reduce plot size.
